# EFFECTS OF CONSERVATIVE VALUES AND AGING KNOWLEDGE ON YOUNG ADULTS’ AGEISM AGAINST OLDER ADULTS

**DOI:** 10.1093/geroni/igad104.2703

**Published:** 2023-12-21

**Authors:** Joseph Muraira, Erin Hulsman, Christian López, Derek Killingsworth, Kellee Bishop, Jenna Lao, Allyson Coldiron, Michael Barnett

**Affiliations:** University of Texas at Tyler, Tyler, Texas, United States; University of Texas at Tyler, Tyler, Texas, United States; University of Texas at Tyler, Tyler, Texas, United States; University of Texas at Tyler, Tyler, Texas, United States; University of Texas at Tyler, Tyler, Texas, United States; University of Texas at Tyler, Tyler, Texas, United States; University of Texas at Tyler, Tyler, Texas, United States; University of Texas at Tyler, Tyler, Texas, United States

## Abstract

Ageism – the stereotypes, prejudice, and discrimination of individuals solely based on age – is an unfortunately prevalent phenomenon for older adults to experience from young adults, and increased ageism among young adults has been associated with more conservative values and beliefs. Previous interventions to reduce ageism in young adults have focused on increasing knowledge about aging and contact between generations, since greater aging knowledge is associated with decreased ageism. However, recent studies identify multiple forms of ageism, including negative ageism (e.g., beliefs about older adults’ lack of cognitive and physical ability) and positive ageism (e.g., stereotypes of older people as warm and reliable). This study therefore examined the relationships between young adults’ conservative values, aging knowledge, and both negative and positive ageism about older adults. Participants were young adults (N = 459), ages 18 to 25 years (M = 20.10, SD = 1.76), who completed the Relating to Older People Evaluation, Social and Economic Conservatism Scale, and Palmore’s Facts on Aging Quiz in an online survey. Results showed that conservatism was positively associated with negative and positive ageism, while aging knowledge was only negatively associated with negative ageism. Hierarchical regressions found that aging knowledge explained significant variance in negative ageism when controlling for conservatism, but only conservatism explained variance in positive ageism. These findings suggest that interventions including education on aging for young adults may be able to overcome the negative ageism associated with conservative values, but that positive ageism may not be meaningfully addressed by such education-based interventions.

